# Assessment of neuroinflammation in a mouse model of obesity and β-amyloidosis using PET

**DOI:** 10.1186/s12974-016-0700-x

**Published:** 2016-08-31

**Authors:** Anna M. Barron, Masaki Tokunaga, Ming-Rong Zhang, Bin Ji, Tetsuya Suhara, Makoto Higuchi

**Affiliations:** 1 Department of Functional Brain Imaging Research, National Institutes for Quantum and Radiological Science and Technology, 4-9-1 Anagawa, Inage-ku, Chiba-shi, Chiba 263-8555 Japan; 2 Department of Radiopharmaceutics Development, National Institutes for Quantum and Radiological Science and Technology, 4-9-1 Anagawa, Inage-ku, Chiba-shi, Chiba 263-8555 Japan; 3 Neurobiology of Aging and Disease Laboratory, Lee Kong Chian School of Medicine, Nanyang Technological University, 59 Nanyang Drive, Singapore, 636921 Singapore

**Keywords:** Beta amyloid, Translocator protein, Neuroinflammation, Cerebral metabolism, Alzheimer’s disease

## Abstract

**Background:**

Obesity has been identified as a risk factor for cognitive decline and Alzheimer’s disease (AD). The aim of this study was to investigate the effect of obesity on neuroinflammation and cerebral glucose metabolism using PET in a mouse model of β-amyloidosis and determine the relationship between these PET imaging biomarkers, pathogenic changes, and functional outcomes.

**Methods:**

Three-month-old C57BL/J6 mice were fed either a standard (control group) or high-fat diet (obese group) for 3 months and intracerebroventricularly infused with vehicle or human beta amyloid 1-42 (Aβ_42_). We assessed obesity-induced abnormalities in peripheral metabolic indices including adiposity, fasting glucose, and glucose tolerance. Brain glucose metabolism was assessed by ^18^F-FDG PET, and glial activation was assessed using the translocator protein (TSPO) ligand ^11^C-PBR-28. TSPO expression was confirmed by immunohistochemistry of brain sections obtained from scanned mice. The association between inflammatory state and ^11^C-PBR-28 PET signals was characterized by examination of the cytokine expression profile in both the serum and hippocampus by antibody array. Learning and memory performance was assessed in the object recognition task, and anxiety-related behavior was assessed in the elevated plus maze.

**Results:**

Obesity combined with Aβ infusion promoted neuroinflammation and cerebral hypermetabolism, and these signals were significant predictors of learning and memory performance in the object recognition task. In vivo TSPO signals were associated with inflammatory markers including CXCL1, CXCL2, CXCL12, CCL3, CCL5, TIMP-1, G-CSF, sICAM-1, and IL-1ra.

**Conclusions:**

In vivo cerebral metabolism and TSPO signals indicate that obesity can accelerate amyloid-induced inflammation and associated cognitive decline.

## Background

Obesity is a global health issue owing to high intake of refined carbohydrates and saturated fat and increasingly sedentary lifestyles [1]. Growing evidence suggests that obesity at midlife increases the risk of cognitive decline and Alzheimer’s disease (AD) in later life [2–4]. AD pathogenesis, which begins decades prior to the onset of symptoms [5, 6], is characterized by the accumulation of the toxic beta amyloid (Aβ) peptides, formation of neurofibrillary tangles, chronic inflammation, and neuronal loss [5, 7]. Numerous imaging modalities including structural and functional magnetic resonance imaging (MRI) and positron emission tomography (PET) assessment of cerebral glucose metabolism and amyloid burden have proven diagnostically useful in mild cognitive impairment (MCI) and AD [8]. However, the relationship between imaging biomarkers and early midlife pathogenic changes leading to AD, such as obesity, has remained elusive. Since obesity-driven inflammation and metabolic impairment have been identified as important mechanisms accelerating AD pathogenesis and functional impairment [9–13], they may represent potential early-stage imaging markers of cognitive decline and AD risk.

Neuroinflammation has been identified as a contributor to both the initiation and progression of AD. Accumulation of soluble Aβ is suggested to induce inflammatory processes in AD, activating glial cells and potentiating the production of proinflammatory cytokines [14]. In obesity, chronic systemic inflammation triggers neuroinflammation, thereby exacerbating Aβ-induced degenerative cascades and AD progression [15]. The mitochondrial translocator protein (TSPO) is markedly upregulated in activated glia in inflammatory disease including AD, believed to play a critical role in mediating glial responses to AD pathogenesis. In vitro studies using transgenic mouse models of AD indicated that TSPO expression is linked with neuronal toxicity and death [16, 17]. ^11^C-PBR-28 is a second-generation TSPO ligand with high affinity and penetration of the blood-brain barrier compared to the prototypic first-generation TSPO ligand, ^11^C-PK-11195 [18]. Previous studies have demonstrated the usefulness of ^11^C-PBR-28 in subjects with AD [19] and multiple sclerosis [20] and in animal models of aging [21] and cerebral ischemia [22]. However, the detection of early-stage inflammatory changes associated with obesity-related cognitive decline and AD risk has not been investigated using TSPO ligands.

Cerebral glucose metabolism measured by ^18^F-fluoro-2-deoxy-d-glucose-PET (FDG PET) is widely used in AD as an index of neuronal loss [23]. Although cerebral metabolic changes prior to the development of neuronal loss in AD remains poorly understood, emerging evidence suggests that hypometabolism may be preceded by a hypermetabolic phase [24–27]. It is unclear if this early hypermetabolic stage reflects neuronal hyperactivity or inflammatory changes or if the hyperactivity is a cause (e.g., activity-driven Aβ deposition) or result of AD pathogenesis (e.g., Aβ-induced seizure activity or inflammation) [26, 27]. Transgenic mouse models of amyloidosis which do not develop neuronal loss may be useful tools for investigating metabolic changes associated early stages of Aβ pathogenesis [28–31]. However, use of ^18^F-FDG PET in transgenic animal models may be hampered by the overexpression of amyloid precursor protein (APP) and non-Aβ fragments of APP, which are known to modify synaptic and metabolic activity [28–31]. To circumvent this, here, we have used Aβ-infusion to investigate interactions between Aβ- and obesity-driven inflammatory and metabolic abnormalities in vivo using ^11^C-PBR-28 and ^18^F-FDG PET, respectively, and determine the relationship between these imaging biomarkers, underlying pathogenic changes and functional outcomes.

## Methods

### Animals and treatments

Three-month-old male C57Bl/J mice were purchased (Clear, Japan) and maintained at the National Institute of Radiological Sciences vivarium facilities with food and water available ad libitum. Beginning at 3 months of age, mice were randomly assigned to groups (*n* = 6/group) and maintained on a standard diet (10 % kCal fat; Research Diets D12450B Harlan Teklad, Indianapolis, IN) or a high-calorie, high-fat diet (60 % kCal fat; Research Diets D12492, Harlan Teklad) for a period of 3 months. Body weights were monitored weekly, and fasting blood glucose was monitored fortnightly.

After 1 month of feeding, mice were anesthetized with isoflurane (3 % *v*/*v* for induction, 1.5 % *v*/*v* for maintenance), positioned in a stereotaxic apparatus and 0.9 % saline applied to the eyes. The scalp was shaved and cut, the skull exposed, and adhering tissue was removed with acetone. A cannula (Brain Infusion Kit 3, Alzet) was implanted in the left ventricle at the following coordinates: +1.0 medial/lateral, −0.3 anterior/posterior, −2.5 dorsal/ventral. The cannula was fixed to the skull using dental cement and connected to a mini-osmotic pump (Model 1002, Alzet) that was filled with either vehicle (250 μg/mL high-density lipoprotein (HDL) in 4 mM HEPES with 2.5 % DMSO) or 120-μM oligomeric Aβ-42 [32]. Oligomeric Aβ-42 was prepared by solubilizing synthetic human Aβ-42 (Peptide Institute) to 1 mM in hexafluoroisopropanol, then drying under vacuum in a SpeedVac. The peptide film was then resuspended in DMSO to 5 mM and diluted in 4 mM HEPES containing 250 μg/mL HDL (Millipore) to a final concentration of 120 μM. Pumps were partially coated with paraffin to adjust the infusion rate to 3 μL/day for 1 month, then the filled pumps were incubated in sterile phosphate-buffered saline (PBS) at 37 °C for 40 h prior to implantation under the dorsal skin on the back. The incision site on the scalp was closed with suture, and mice were administered buprenorphrine (0.05 mg/kg i.p., Henry Schein Inc.) post-operatively for analgesia. One spontaneous death occurred in the 8 weeks post-surgery treatment duration (obese + Aβ group).

All experimentation was carried out in strict accordance with the recommendations in the Guide for the Care and Use of Laboratory Animals of the National Institutes of Health and was approved by the Institutional Animal Care and Use Committee of the National Institutes for Quantum and Radiological Science and Technology, Japan.

### Glucose measurements

Fasting blood glucose was assessed using a Nipro Freestyle Glucometer (Nipro Diagnostics, Florida, USA) from the whole blood collected via the tail vein while the mouse was under isofluorane general anesthesia. Mice were fasted overnight for 16 h prior to sample collection. Mice were fasted at baseline (time 0), 1, 2 months and sacrificed for assessment of blood glucose levels. Mice were additionally fasted overnight at 2.5 months for the ^18^F-FDG PET scans and again for 2 days later for the glucose tolerance test. For the glucose tolerance test, baseline glucose levels were measured, then fasted mice were injected with 2 mg glucose/g body weight (i.p.), and blood glucose was measured from the whole blood collected via the tail vein 30, 60, and 120 min after injection.

### In vivo PET imaging

TSPO signals were assessed by PET using ^11^C-PBR-28, which was prepared according to previously published methods [33]. The specific activity of the end product was 80.7 ± 14.7 GBq/μmol and the radiochemical purity exceeded 95 %. ^18^F-FDG was purchased from Nihon Medi-Physics Co. LTD (Tokyo, Japan). Mice were fasted prior to ^18^F-FDG PET scans, and blood glucose levels were assessed at the completion of scan.

**Fig. 1:**
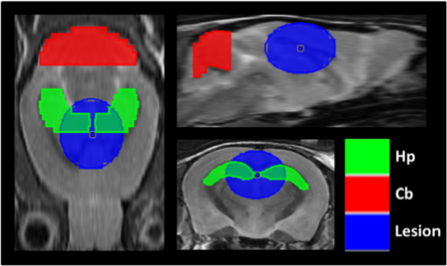
Anatomical localization of volumes of interest (VOIs). *Horizontal*, *sagittal*, and *coronal* MRI slices of the mouse brain indicating the hippocampal (Hp), cerebellar (Cb), and lesion-site (Lesion) VOIs used for PET quantification

Mice were anesthetized with 1.5 % (*v*/*v*) isoflurane and administered ^11^C-PBR-28 (27.9 ± 0.9 MBq) or ^18^F-FDG (17.6 ± 0.2 MBq) via a tail vein catheter while in a microPET Focus 220 animal scanner (Siemens Medical Solutions). Simultaneous with radioligand injection, 3D list mode data acquisitions were started and lasted 60 min. Summation images were reconstructed with maximum a posteriori reconstruction (40–60 min for ^11^C-PBR-28 and 20–60 min for ^18^F-FDG), and dynamic images were reconstructed with filtered back-projection using a 0.5-mm Hanning filter, which were used for quantification of standardized uptake values (SUVs) and percentage of injected dose per tissue volume (%ID/g). Volumes of interest (VOIs) were placed at the infusion site and in the hippocampal and cerebellar regions with reference to the MRI template (Fig. 1). Then, dynamic PET images were merged to the MRI template, and the tracer uptake in each VOI was estimated as %ID/g for ^11^C-PBR-28 (40–60 min) and SUV for ^18^F-FDG (20–60 min). PET image analysis described above was performed with PMOD image analysis software (version 3.4; PMOD Technologies, Inc., Zurich, Switzerland).

### Behavioral assessment

Short-term working memory was assessed in the object recognition task. Mice were placed in the centre of an open field (50 × 50 × 50 cm) and allowed to freely explore for 3 min (habituation trial). Following a 2-h interval, mice were again placed in the centre of the maze between two identical objects and allowed to explore freely for 6 min (training trial). The frequency of the left and right object explorations were scored to test for spatial bias. After a 24-h inter-trial interval, one object was replaced with a novel object and the mouse was allowed to freely explore for 10 min (probe trial). The duration of novel (*D*_n_) and familiar (*D*_f_) object explorations were recorded and used to calculate the percent recognition (*D*_n_/(*D*_n_ + *D*_f_)).

Anxiety-related behavior was assessed in the elevated plus maze (EPM). Mice were placed in the center of a plus-shaped maze (25 cm × 8 cm) with two open arms and two closed arms elevated 60 cm from the ground. The closed arms were surrounded by 24-cm walls. Behavior was recorded in a 5-min period via overhead digital video and analyzed using SMART Automated Recording (Panlab). The total duration spent in the open arm was calculated as an index of anxiety-related behavior.

### Tissue collection and preparation

Mice were fasted for 16 h prior to sacrifice then deeply anesthetized (50 mg sodium pentobarbital/kg body weight, i.p.). Blood was collected via cardiac puncture and fasting blood glucose assessed. Serum was collected from the remaining blood and stored at −80 °C. Mice were then intracardially perfused with ice-cold, sterile saline, and one hemibrain was submersion fixed in 4 % paraformaldehyde/0.1 M PBS (pH 7.4) for 24 h for immunohistochemistry. The hippocampus was rapidly dissected from the remaining hemibrain and snap frozen on liquid nitrogen. Retroperitoneal and subcutaneous fat pads were collected at necropsy and weighed as an indicator of visceral adiposity and body composition.

### Immunohistochemistry

Fixed hemibrains were cryoprotected in 30 % sucrose, frozen, and sectioned in the horizontal plane at 20 μM using a cryostat. Sections were soaked in PBS for 5 min, then immunolabeled with antibodies directed against TSPO (rabbit monoclonal, 1:1000; Abcam), glial fibrillary acidic protein (GFAP; rat monoclonal, 1:1000 dilution, 2.2B10, Zymed), or anti-ionized calcium-binding adaptor molecule-1 (IBA-1; rabbit polyclonal, 1:1000 dilution, WAKO). Non-specific staining was blocked in blocking reagent (PerkinElmer) for 1 h at room temperature, then immunolabeled overnight at 4 °C with primary antibodies. Sections were then washed three times in PBS for 5 min and then incubated with anti-rabbit or anti-rat IgG biotin (1:1000) for 1 h at room temperature. Immunoreactivity was visualized using tetramethylrhodamine-labeled tyramide signal amplification (PerkinElmer). Sections were mounted with VectaSheild (Vector Laboratories), and coverslips were sealed with enamel. Photomicrographs were captured with a fluorescence microscope/digital camera (BZ-X700, Keyence) at ×20 magnification and then thresholded at a predetermined, constant value using NIH Image 1.61 to create a binary image identifying positive and negative immunolabeling and the intensity assessed.

### Cytokine antibody microarray

Mouse cytokine antibody arrays (cytokine array panel A, Proteome Profiler, R&D systems, Minneapolis, USA) were used to analyze cytokine expression profiles in serum and hippocampal samples according to the manufacturer’s instructions. Frozen hippocampus samples were homogenized by bead cell disrupter (Micro Smash) in PBS containing complete ULTRA protease inhibitors (Roche). A digital imaging system (Chemidoc, Bio-Rad) was used to detect chemiluminescent signals which were analyzed using ImageJ software (NIH). Measured cytokines included the following: complement component 5 (C5a), chemokine (C–C motif) ligand 1 (CCL1), CCL2, CCL3, CCL4, CCL5, CCL-11, CCL12, CCL17, chemokine (C–X–C motif) ligand 1 (CXCL1), CXCL2, CXCL9, CXCL10, CXCL11, CXCL12, CXCL13, granulocyte colony-stimulating factor (G-CSF), granulocyte macrophage colony-stimulating factor (GM-CSF), intracellular adhesion molecule 1 (ICAM-1), interferon gamma (IFN-γ), interleukin-1α (IL-1α), IL-1β, IL-1ra, IL-2, IL-3, IL-4, IL-5, IL-6, IL-7, IL-10, IL-12p70, IL-13, IL-16, IL-17, IL-23, IL-27, macrophage colony-stimulating factor (M-CSF), tissue inhibitor of metalloproteinase 1 (TIMP-1), tumor necrosis factor alpha (TNF-α), and triggering receptor expressed on myeloid cells 1 (TREM-1).

### Statistical analysis

Parametric data was analyzed by one-way analysis of variance (ANOVA) or linear mixed model analysis using the Statistical Package for Social Sciences (SPSS: version 11.5; SPSS Inc., IL, USA). Data were analyzed using the information-theoretic approach, using the Akaike information criterion (AIC) model selection process and linear regression with Bonferroni adjustment for multiple analyses. All data are presented as mean ± standard error of the mean (SEM). Significance was set at a threshold of *p* < 0.05.

## Results

**Fig. 2:**
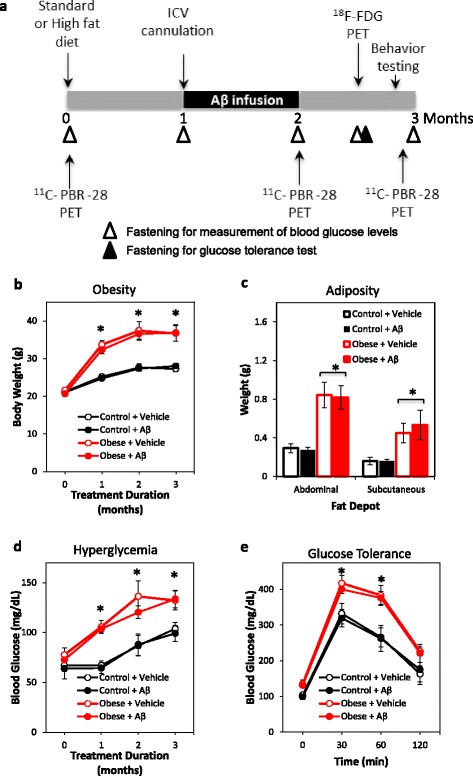
Treatment schedule and validation of the obesity model. **a** Feeding with regular (control group) or high-fat diet (obese groups) began at month 0, at which time baseline TSPO signals were also assessed in vivo via ^11^C-PBR-28 PET. ICV cannulas for infusion of vehicle or Aβ were implanted at month 1 and infusion was continuous until month 2. ^11^C-PBR-28 PET was repeated at months 2 and 3. ^18^F-FDG PET was assessed at month 2.5. Behavior testing was carried out at month 3, prior to the final ^11^C-PBR-28 PET scan. Fasting time points are indicated by *arrowheads*. Tissues were collected 3 months after the initiation of regular or high-fat diet feeding. **b** Body weight in mice across the 3-month treatment duration, with significant obesity observed in high-fat fed groups from 1 month. Month 0: *F* = −11.59, *p* = 0.38; month 1: *F* = 11.05, *p* < 0.001; month 2: *F* = 10.46, *p* = 0.001; month 3: *F* = 9.98, *p* = 0.003. **c** Abdominal retroperitoneal fat pad and subcutaneous fat pad weight in mice, confirming increased adiposity in high-fat fed groups. *F* = 9.21, *p* = 0.01. **d** Fasting blood glucose across the 3-month treatment duration, with significant hyperglycemia observed in high-fat fed groups from 1 month. Month 0: *F* = 0.65, *p* = 0.59; month 1: *F* = 19.89, *p* < 0.001; month 2: *F* = 5.69, *p* = 0.006; month 3: *F* = 4.93, *p* = 0.01. **e** Glucose tolerance test in control and obese mice infused with vehicle or Aβ. Obese mice exhibited significant glucose intolerance. Baseline: *F* = 4.99, *p* = 0.01; 30-min post-glucose challenge: *F* = 4.74, *p* = 0.01; 60-min post-glucose challenge: *F* = 5.67, *p* = 0.006. Data presented as mean ± SEM. *p < 0.05 relative to matched time point control mice

In this study, we investigated obesity-driven inflammatory and metabolic abnormalities in an Aβ-infusion model of β-amyloidosis using PET. Obesity and hyperglycemia were evident in the high-fat fed mice after 1 month of feeding, accompanied by significantly increased abdominal and subcutaneous fat deposition and impaired glucose tolerance at sacrifice (Fig. 2b–d). Mice were infused with either vehicle or oligomeric human Aβ_42_ 1 month after feeding initiation, once obesity was already evident in high-fat fed groups (Fig. 2a). In vivo TSPO signals were assessed at baseline, 2, and 3 months after feeding initiation via ^11^C-PBR-28 PET, while cerebral metabolism was assessed 2.5 months after feeding initiation via ^18^F-FDG PET (Fig. 2a). Learning and memory and anxiety-related behaviors were assessed 3 months after feeding initiation, prior to the final ^11^C-PBR-28 PET scan (Fig. 2a).

### Longitudinal in vivo PET imaging of obesity and Aβ-triggered inflammation using ^11^C-PBR-28

**Fig. 3:**
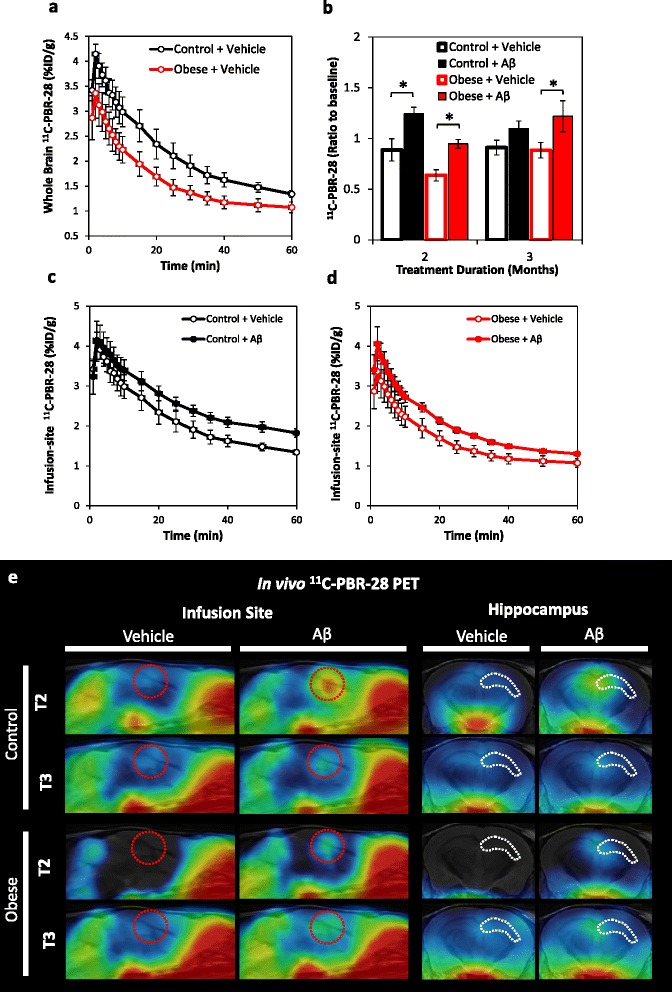
Aβ-triggered inflammatory signals detected by in vivo PET with ^11^C-PBR-28. **a** Whole-brain time activity curves in control versus obese mice 2 months post-treatment. Obesity significantly reduced brain ^11^C-PBR-28 uptake, most likely reflecting altered whole body ^11^C-PBR-28 distribution. **b** Group comparison of ^11^C-PBR-28 accumulation at the infusion site at 2 and 3 months post-treatment (relative to individual baseline accumulation). Obesity combined with Aβ infusion synergistically increased radioligand accumulation at the infusion site, peaking at 2 months, but persisting at 3 months. **c** Time activity curves at the infusion site of vehicle versus Aβ-infused mice control mice at 2 months. **d** Time activity curves at the infusion site in vehicle versus Aβ-infused obese mice at 2 months. **e** Sagittal and coronal PET images in control and obese mice infused with vehicle or Aβ at 2 (T2) and 3 months post-treatment (T3). A marked increase in radioactivity was observed surrounding the Aβ infusion site in the lateral ventricle (indicated by *dashed red outline)* extending into the dorsal hippocampus (indicated by *white dashed outline*). Images were generated by summation of dynamic data between 40 and 60 min after injection of ^11^C-PBR-28 and were overlaid on the MRI template. *p < 0.05

To examine the effect of obesity and β-amyloidosis on TSPO inflammatory signals, ^11^C-PBR-28 uptake was compared at baseline, 2-, and 3-month treatment time points (Fig. 3). Obesity can alter the whole body distribution of ^11^C-PBR-28; therefore, the brain uptake expressed as %ID/g may result in an underestimation of TSPO signals in obese groups (Fig. 3a). Aβ significantly increased ^11^C-PBR-28 signals at the infusion site and extending into the dorsal hippocampus in both control and obese groups at the 2-month treatment time point (*F* = 10.94; *p* = 0.001; Fig. 3b–e). At 3 months, increased ^11^C-PBR-28 signals persisted in obese but not in control mice infused with Aβ (*F* = 3.80, *p* = 0.03; Fig. 3b, e).

**Fig. 4:**
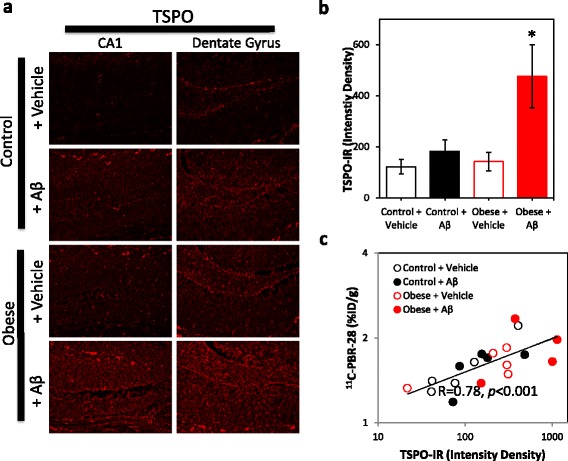
Obesity and Aβ increase hippocampal TSPO immunoreactivity. **a** Representative images of TSPO immunoreactivity (*red*) in the CA1 and dentate gyrus regions of the hippocampus. **b** Quantification of TSPO immunoreactivity (TSPO-IR) in the CA1 and dentate gyrus regions of control and obese mice infused with either vehicle or Aβ. Obesity combined with Aβ infusion significantly increased hippocampal TSPO immunoreactivity. **c** Association between hippocampal ^11^C-PBR-28 accumulation at 3 months and TSPO immunoreactivity. Data presented as mean ± SEM. *p* < 0.05

In vivo hippocampal TSPO signals were confirmed in tissues by immunohistochemistry (Fig. 4). Significantly increased TSPO immunoreactivity was observed in the hippocampus of Aβ-infused obese mice (*F* = 4.51, *p* = 0.007; Fig. 4b), and TSPO immunoreactivity correlated with in vivo hippocampal ^11^C-PBR-28 signals (*R* = 0.74, *P* < 0.001; Fig. 4c).

### Obesity and Aβ synergistically increase hippocampal gliosis

**Fig. 5:**
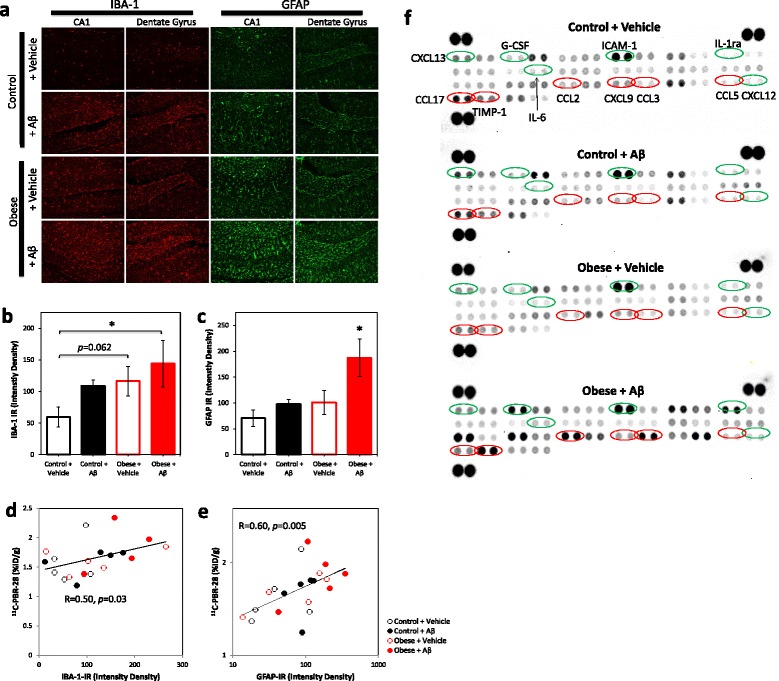
Obesity and Aβ increase hippocampal inflammation. **a** Representative images of IBA-1 (*red*) and GFAP (*green*) immunoreactivity in the CA1 and dentate gyrus regions of the hippocampus. **b** Obesity and Aβ infusion, either alone or in combination, increased IBA-1 immunoreactivity (IBA-1-IR) in the CA1 and dentate gyrus regions of obese and Aβ-infused mice. **c** Obesity combined with Aβ infusion synergistically increased GFAP immunoreactivity (GFAP-IR) in the CA1 and dentate gyrus regions of the hippocampus. **d** Association between hippocampal ^11^C-PBR-28 accumulation at 3 months and GFAP immunoreactivity. **e** Association between hippocampal ^11^C-PBR-28 accumulation at 3 months and IBA-1 immunoreactivity. **f** Representative hippocampal cytokine antibody microarray blots from control + vehicle, control + β, obese + vehicle, and obese + Aβ hippocampal samples. Inflammatory markers identified as significant predictors of ^11^C-PBR-28 signals are *circled in green*. Inflammatory markers significantly correlating with ^11^C-PBR-28 signals are *circled in red*. Data presented as mean ± SEM. *p* < 0.015

To assess whether ^11^C-PBR-28 PET signals at 3 months were associated with gliosis, IBA-1 and GFAP levels were assessed by immunohistochemistry in the hippocampus (Fig. 5a). Obesity combined with Aβ infusion significantly increased IBA-1 immunoreactivity relative to vehicle-treated control mice (*F* = 2.96, *p* = 0.05; Fig. 5b) and increased GFAP immunoreactivity compared to all other groups (*F* = 5.13, *p* = 0.004; Fig. 5c). Hippocampal ^11^C-PBR-28 PET signals significantly correlated with hippocampal IBA-1 (*r* = 0.45, *p* = 0.05; Fig. 5d) and GFAP immunoreactivity (*r* = 0.62, *p* = 0.005; Fig. 5e).

To characterize the association between inflammatory state and ^11^C-PBR-28 PET signals, the cytokine expression profile in both the serum and hippocampus was evaluated by an antibody array (Fig. 5f). Correlation and multiple regression analyses were conducted to examine the relationship between ^11^C-PBR-28 PET signals (%ID/g) in the hippocampus measured at the final time point and potential inflammatory predictors (Table 1). Serum levels of M-CSF and IL-2 and hippocampal levels of CCL5, CCL3, IL-1ra, CCL2, CXCL9, and TIMP-1 significantly positively correlated with ^11^C-PBR-28 PET signals, while hippocampal CCL17 and IL-6 significantly negatively correlated. Hippocampal ^11^C-PBR-28 PET signals were significantly predicted by both serum and hippocampal CXCL12 and CXCL13; serum IL-13, IL-1β, CXCL1, CXCL2, CCL3, CCL5, and TIMP-1; and hippocampal G-CSF, IL-1ra, IL-6, and ICAM-1 as assessed by multiple linear regression analysis, with the predictor variables explaining greater than 99 % of ^11^C-PBR-28 PET signal variability (*F* = 8014.77, *p* < 0.001, *R*^2^ = 0.999; Table 1).

**Table 1:** Multivariate linear regression analysis of serum and hippocampal cytokines associated with in vivo hippocampal ^11^C-PBR-28 uptake assessed by PET

		Cytokine	^11^C-PBR-28 correlation	Multiple regression weights	
				*b*	*β*
Serum	Predictors	TIMP-1	0.075	0.0011**	2.3454
		CXCL1	−0.129	−0.0008**	−1.9194
		CXCL2	0.25	0.0040**	1.6419
		CCL3	−0.057	−0.0026**	−1.5286
		CCL5	0.038	0.0023**	0.8512
		IL-1β	0.053	−0.0012**	−0.6740
		IL-13	0.281	0.0002**	0.5435
		CXCL12	−0.099	0.0002**	0.3181
		CXCL13	0.137	−0.0003**	−0.2792
	Correlates	M-CSF	0.650**		
		IL-2	0.537*		
Hippocampus	Predictors	CXCL13	0.045	−0.0024**	−2.923
		G-CSF	0.069	0.0074**	2.159
		IL-1ra	0.641*	0.0072**	1.550
		IL-6	−0.531*	−0.0001**	−0.518
		CXCL12	0.389	−0.0008**	−0.625
		sICAM-1	−0.245	−0.0008*	−0.474
	Correlates	CCL17	−0.838***		
		CCL3	0.691**		
		CCL2	0.637*		
		CXCL9	0.634*		
		TIMP-1	0.522*		
		CCL5	0.553*		

*Abbreviations*: *b* unstandardized coefficients, *β* standardized coefficients, *TIMP-1* tissue inhibitor of metalloproteinases, *M-CSF* macrophage colony-stimulating factor, *IL-2* interleukin 2, *G-CSF* granulocyte colony-stimulating factor, *IL-ra* interleukin 1 receptor antagonist, *IL-6* interleukin 6, *ICAM-1* intracellular adhesion molecule 1

*p < 0.05; **p < 0.01; ***p < 0.001

### PET imaging of obesity and Aβ-induced effects on cerebral metabolism using ^18^F-FDG PET

**Fig. 6:**
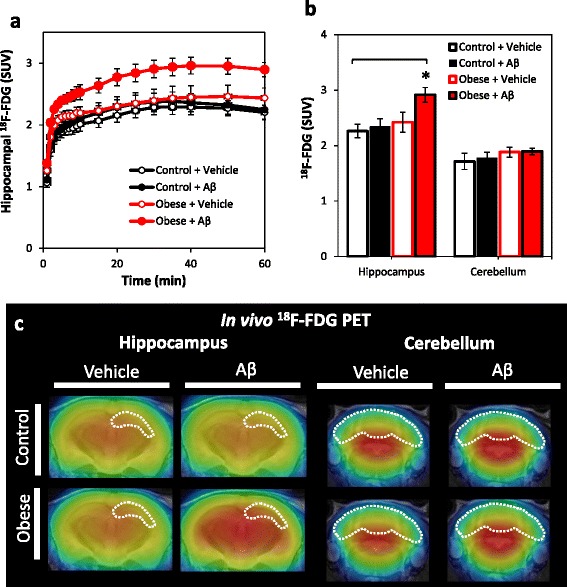
Obesity and Aβ-triggered hyperactive cerebral metabolism detected by in vivo PET with ^18^F-FDG. **a** Hippocampal ^18^F-FDG time activity curves in control versus obese mice. **b** Group comparison of cerebral metabolism assessed by ^18^F-FDG accumulation in the hippocampus and cerebellum. Obesity combined with Aβ infusion synergistically increased radioligand accumulation in the hippocampus but not in the cerebellum. **c** Representative coronal ^18^F-FDG PET images in vehicle or Aβ-infused control (*top panel*) and obese mice (*bottom panel*). A marked increase in radioactivity was observed in the hippocampus (indicated by *dashed line*, *left panel*) and underlying limbic structures. No effect of treatment was observed on radioactivity in the cerebellum (indicated by *white dashed line*, *right panel*). Images were generated by summation of dynamic data between 0 and 90 min after injection of ^18^F-FDG and were overlaid on the MRI template. *p < 0.05

To examine the effect of obesity and β-amyloidosis on cerebral metabolism, ^18^F-FDG uptake was examined at the 2.5-month treatment time point (Fig. 6). A significant increase in ^18^F-FDG uptake in the hippocampus but not the cerebellum was observed in Aβ-infused obese mice (hippocampus: *F* = 3.99; *p* = 0.02; cerebellum: *F* = 2.23, *p* = 0.12; Fig. 6). Hippocampal ^18^F-FDG SUVs were not significantly associated with markers of adiposity or glucose metabolism (data not shown).

### ^11^C-PBR-28 and ^18^F-FDG PET signals predict learning and memory impairment

**Fig. 7:**
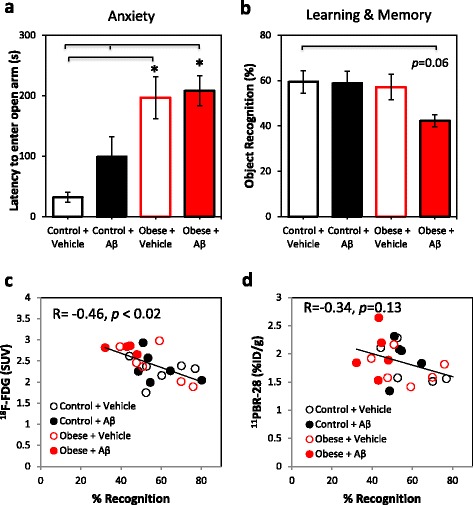
Association between functional performance in anxiety and learning and memory tasks and ^18^F-FDG and ^11^C-PBR-28 PET signals. **a** Aβ infusion and obesity increased anxiety-related behavior in the elevated plus maze. **b** A trend towards impaired learning and memory performance in Aβ-infused obese mice approached significance in the object recognition task. **c** Hippocampal ^18^F-FDG signals significantly negatively correlated with learning and memory performance in the object recognition task. **d** No significant correlation between hippocampal ^11^C-PBR-28 signals and learning and memory performance was observed, although both ^18^F-FDG and ^11^C-PBR-28 were significant predictors of learning and memory performance as assessed by multiple regression analysis. *p* < 0.05

At the 3-month treatment time point, the effect of obesity and Aβ infusion on anxiety-related behavior and learning and memory performance was assessed in the EPM and the object recognition tasks, respectively. Obesity significantly increased in anxiety-related behavior, resulting in increased latency to enter the open arm compared to the control mice, while obesity combined with Aβ-infusion increased anxiety-related behavior compared to both vehicle and Aβ-infused control groups (*F* = 11.54, *p* < 0.001; Fig. 7a). Treatment did not affect locomotor activity, with no significant effect of treatment on total distance moved in the EPM (*F* = 0.11, *p* = 0.96). Although a significant effect of treatment was observed on object recognition performance (*F* = 10.32, *p* = 0.02), no significant group differences were observed by post hoc analysis, with the effect of obesity combined with Aβ infusion approaching significance (*p* = 0.06; Fig. 7b). Interestingly, ^18^F-FDG SUVs significantly correlated with anxiety-related behavior in the elevated plus maze (*r* = 0.48, *p* = 0.02) and negatively correlated with learning and memory in the object recognition task (*r* = −0.46, *p* = 0.02; Fig. 7c). Hippocampal ^18^F-FDG and ^11^C-PBR-28 PET signals at 3 months significantly predicted learning and memory performance but not anxiety-related behavior as assessed by multiple linear regression analysis, with the predictor variables explaining approximately 40 % of the variability in object recognition performance (*F* = 6.06, *p* = 0.009, *R*^2^ = 0.39). Both ^18^F-FDG (*B* = −19.55, *β* = −0.62, *p* = 0.005) and ^11^C-PBR-28 (*B* = −17.59, *β* = −0.45, *p* = 0.03) significantly contributed to the model.

## Discussion

Using ^11^C-PBR-28-PET, we detected elevated TSPO associated with activated astrocytes and microglia resulting from Aβ-induced inflammation. The Aβ-induced inflammatory response was transient, with TSPO signals no longer significantly elevated at the completion of the experiment. However, the magnitude and duration of the Aβ-induced inflammatory response was exacerbated by obesity, even though obesity alone did not increase TSPO inflammatory signals. Previous studies using TSPO as a marker of inflammation in transgenic mouse models of β-amyloidosis have also reported elevated TSPO signals detected by PET, although only in aged mice at late stages of neuropathology [17, 34, 35]. What remains unclear is to what extent TSPO expression represents deleterious versus trophic glial responses. While many studies have focused on TSPO as a marker of deleterious gliosis, reporting good correlations between microglial markers and TSPO PET signals [36], some evidence suggests that TSPO-positive glial activation may be associated with trophic glial functions including Aβ clearance [37]. To address this, in the current study, the systemic and hippocampal cytokine profile was examined to determine which specific inflammatory factors were associated with in vivo TSPO signals. Serum levels of numerous obesity-linked chemokines promoting adipocyte proliferation (TIMP-1 [38]) and adipose macrophage infiltration (CXCL1 [39, 40], CXCL2 [41], CXCL12 [42], CCL3 [43], and CCL5 [44]) were found to be significant predictors of in vivo hippocampal TSPO signals. Interestingly, in the hippocampus, levels of G-CSF [45, 46], ICAM-1 [47], and CXCL12 [48] and IL-1ra [49], which have been identified as inflammatory factors important in the regulation of amyloid clearance, significantly predicted in vivo hippocampal TSPO signals. Identification of the TPSO-associated inflammatory profile is necessary in order to understand the specific glial functions associated with TSPO signals, which is essential for the validation of TSPO as an effective biomarker of β-amyloidosis in AD.

In contrast to clinical observations in advanced AD, in the current study, β-amyloidosis and cognitive impairment were associated with increased cerebral metabolism as assessed by ^18^F-FDG PET. Numerous other studies have also associated hypermetabolism with early stages of amyloid pathology in transgenic mouse models of β-amyloidosis [28–31]. Luo et al. reported hypermetabolism in young Tg2576 mice, prior to the development of plaques [29]. Previous studies have suggested hypermetabolism may result from increased gliosis in the absence of neuronal loss; however, we observed poor overlap between regions of hyperactivity and TSPO signals and no association between cerebral metabolism and markers of gliosis. Our findings suggest that hypermetabolic changes cannot be explained by gliosis alone. Multiple metabolic disturbances like altered glucose uptake and glycolytic metabolism has been mechanistically implicated in β-amyloidosis [50] and Aβ-induced astrocytic and neuronal hyperactivity and seizure activity [51–54]. In very young AD mutation carriers, approximately 25 years before the expected onset of symptoms, region-specific hypermetabolism is observed prior to the development of hypometabolism [24]. Likewise, amyloid deposition has been found to be associated with hypermetabolism in normal aging and MCI [25–27]. Functional MRI (fMRI) studies have also demonstrated increased brain activation in normal controls with amyloid deposition [55], in MCI [56–60] and in subjects at genetic risk for AD [61–63]. Cerebral hypermetabolism may be induced in early stages of β-amyloidosis, reflecting Aβ-induced pathogenic mechanisms including glial and neuronal hyperactivity, preceding neuronal loss.

In contrast to our previous findings [13], in the current study, obesity alone did not result in a significant deficit in learning and memory performance in the object recognition task. The lack of any deficit in obese mice in the current study is likely a consequence of poor performance of vehicle-treated control mice, which exhibited around 60 % recognition. From our previous studies, we expect the performance of control mice to be around 75 % in this task. We hypothesize that the reduced performance observed in control mice in the current study may be the consequence of repeated interventions including the pump and cannula implantation surgery and imaging experiments, with the stress on the procedures potentially impairing cognitive performance. This reduced the overall sensitivity of the object recognition test, making it difficult to detect impairment resulting from the experimental manipulations.

Interestingly, we did not observe any significant effect of obesity alone on markers of neuroinflammation, cerebral metabolism, or cognitive function. Findings from previous studies are mixed, with some studies reporting increased cortical or hippocampal markers of inflammation in high-fat fed, obese mice [64–66]; others report modest region-specific effects or no detectable effect on the hippocampus [67, 68]. These differences may reflect differences in the severity of metabolic symptoms between mouse strains, diet composition (e.g., cholesterol and caloric and macronutrient content), and duration of treatment exposure. However, this may not be detectable by PET due to methodological issues.

In the current study, obesity induced some limitations including not only logistical and image quality issues due to scatter and attenuation but also in validation of analysis models where large differences in body weight affect radioligand distribution throughout the body. SUV has been widely validated as an outcome measure for ^18^F-FDG uptake adjusting for differences in body weight. However, hyperglycemia is associated with reduced cerebral ^18^F-FDG uptake due to increased competition to cross the blood-brain barrier, likely resulting in an underestimation of cerebral metabolism in obese groups [69–71]. In the case of ^11^C-PBR-28, systemic inflammation and increased TSPO binding in peripheral tissues of obese animals [72] may also have significant impacts on brain uptake. These factors are likely contributors to the reduction in in vivo brain ^11^C-PBR-28 signals observed in obese mice; however, good agreement was observed between in vivo TSPO signals and immunoreactivity, validating the %ID/g for quantifying TSPO in living brains.

## Conclusions

Obesity combined with Aβ infusion was found to increase both TSPO-PET signals and cerebral glucose metabolism. These signals can predict learning and memory performance. In vivo TSPO signals were confirmed by ex vivo analyses and correlated with inflammatory markers of hippocampal gliosis and serum levels of cytokine and chemokines. In contrast, since hypermetabolic regions did not directly correspond to regions of increased TSPO signals, it may reflect early pathogenic effects of Aβ toxicity on neurons rather than inflammation. These findings demonstrate the potential usefulness of TSPO and cerebral glucose metabolism for the investigation of interactions between midlife lifestyle factors and Aβ amyloidosis with the aid of neuroimaging.

## Abbreviations

Aβ: Beta amyloid
AD: Alzheimer’s disease
AIC: Akaike information criterion
ANOVA: Analysis of Variance
C5a: Complement component 5
CCL: Chemokine (C–C motif) ligand
CXCL: Chemokine (C–X–C motif) ligand
EPM: Elevated plus maze
FDG: Fluoro-2-deoxy-d-glucose
G-CSF: Granulocyte colony-stimulating factor
GFAP: Glial fibrillary acidic protein
GM-CSF: Granulocyte macrophage colony-stimulating factor
HDL: High-density lipoprotein
IBA-1: Ionized calcium-binding adaptor molecule 1
ICAM-1: Intracellular adhesion molecule 1
IFN-γ: Interferon gamma
IL: Interleukin
M-CSF: Macrophage colony-stimulating factor
MCI: Mild cognitive impairment
MRI: Magnetic resonance imaging
PBS: Phosphate-buffered saline
PET: Positron emission tomography
SEM: Standard error of mean
SUV: Standardized uptake value
TIMP-1: Tissue inhibitor of metalloproteinase 1
TNF-α: Tumor necrosis factor alpha
TSPO: Translocator protein
TREM-1: Triggering receptor expressed on myeloid cells 1
VOI: Volume of interest
%ID/g: Percentage of injected dose per tissue volume
